# Comparing the use of traditional and agile development methodologies in a clinical trials environment

**DOI:** 10.1186/1745-6215-14-S1-P65

**Published:** 2013-11-29

**Authors:** Mary Rauchenberger, Emma Little, Carlos Diaz Montana

**Affiliations:** 1 MRC Clinical Trials Unit, London, UK

## 

Computer systems used for clinical trials need to have documented evidence that they are validated systems. This is a formal process that assures users (and regulatory inspectors) that the system is fit for purpose. At MRC CTU, we have successfully met this requirement for the past ten years using the traditional waterfall method of software development, which produces agreed documentation at each stage of the life cycle, in sequential order. However, we often find that requirements for a trial can change during development, which adversely affects timelines and increases the documentation burden. Agile development methods are designed to produce smaller quicker deliverables, which is attractive, but are typically less formal in the documentation produced, which present a challenge when working in a regulated environment. We explore the advantages and disadvantages of several flavours of these methodologies, assembling best practice to optimise efficiency and compliance, and describe the implementation of this methodology in some of our newer trials.

